# Cocaine augments neuro-inflammation via modulating extracellular vesicle release in HIV-1 infected immune cells

**DOI:** 10.1186/s12977-021-00570-4

**Published:** 2021-09-16

**Authors:** Manojkumar Narayanan, Rutuja Kulkarni, Shuxian Jiang, Fatah Kashanchi, Anil Prasad

**Affiliations:** 1 Division of Experimental Medicine, Beth Israel Deaconess Medical Center, Harvard Medical School, Boston, MA 02215 USA; 2 Laboratory of Molecular Virology, School of Systems Biology, George Mason University, Manassas, VA 20110 USA

**Keywords:** Extracellular vesicles, Cocaine, HIV-1, Macrophages, Neuroinflammation

## Abstract

**Background:**

Extracellular Vesicles (EV) recently have been implicated in the pathogenesis of HIV-1 syndromes, including neuroinflammation and HIV-1 associated neurological disorder (HAND). Cocaine, an illicit stimulant drug used worldwide is known to exacerbate these HIV-1 associated neurological syndromes. However, the effects of cocaine on EV biogenesis and roles of EVs in enhancing HIV-1 pathogenesis are not yet well defined.

**Results:**

Here, we investigated the effects of cocaine on EV biogenesis and release in HIV-1 infected immune cells and explored their roles in elicitation of neuroinflammation. We found that cocaine significantly augmented the release of EVs from uninfected and HIV-1 infected T-cells, DCs and macrophages. Further analysis of the molecular components of EVs revealed enhanced expression of adhesion molecules integrin β1 and LFA-1 in those EVs derived from cocaine treated cells. Intriguingly, in EVs derived from HIV-1 infected cells, cocaine treatment significantly increased the levels of viral genes in EVs released from macrophages and DCs, but not in T-cells. Exploring the molecular mechanism to account for this, we found that DCs and macrophages showed enhanced expression of the cocaine receptor Sigma 1-Receptor compared to T-cells. In addition, we found that cocaine significantly altered the integrity of the RNA-induced silencing complex (RISC) in HIV-1 infected macrophages and DCs compared to untreated HIV-1 infected cells. Characterizing further the molecular mechanisms involved in how cocaine increased EV release, we found that cocaine decreased the expression of the interferon-inducible protein BST-2; this resulted in altered trafficking of intracellular virus containing vesicles and EV biogenesis and release. We also observed EVs released from cocaine treated HIV-1 infected macrophages and DCs enhanced HIV-1 trans-infection to T-cells compared to those from untreated and HIV-1 infected cells. These EVs triggered release of proinflammatory cytokines in human brain microvascular endothelial cells (HBMECs) and altered monolayer integrity.

**Conclusions:**

Taken together, our results provide a novel mechanism which helps to elucidate the enhanced prevalence of neurological disorders in cocaine using HIV-1 infected individuals and offers insights into developing novel therapeutic strategies against HAND in these hosts.

**Supplementary Information:**

The online version contains supplementary material available at 10.1186/s12977-021-00570-4.

## Background

Despite significant advances in combatting the AIDS epidemic, HIV-1 infection remains a global health problem due to lack of an effective vaccine and frequent treatment failure [1, 2]. Importantly, the prevalence of HIV-1 associated neurological disorders has significantly increased due to the difficulty of efficient delivery of anti-HIV drugs to the central nervous system (CNS) and the effects of substance abuse [3–6].

Recent studies indicate a strong correlation between chronic cocaine abuse and HIV-1 associated neurological complications [3]. Cocaine is a commonly used illicit drug and prominently linked to HIV-1 infection and spread [7–19]. Previous research has shown that cocaine enhances HIV-1 replication in various cell types, alters the immune response by regulating the secretion of cytokines and expression of their receptors and accelerates the decline of CD4+ T-cell counts [7, 9, 11–14, 18, 19]. Cocaine treated human brain microvascular endothelial cells (HBMECs) showed increased release of proinflammatory mediators which induced neuroinflammation and altered the integrity of the blood brain barrier (BBB) [3, 20]. Cocaine also enhanced the expression of adhesion molecules in HBMECs, which resulted in adhesion and transmigration of leukocytes across the endothelial cell monolayer [3]. Our recent studies revealed that cocaine can induce overexpression of co-receptors and significantly augment HIV-1 transfer from DCs to T-cells by increasing an infectious synapse formation. Further, we observed that cocaine altered intracellular trafficking of HIV-1 by enhancing its co-localization to multi vesicular bodies (MVBs) and decreasing its degradation at the phagolysosomal complex [21]. Recently, Carone *et al.* have demonstrated increased extracellular vesicles (EV) release in glioblastoma cells upon cocaine treatment, further indicating a role for the drug in regulation of EV release [22]. This highlights the importance of exploring the effects of cocaine on intracellular trafficking of HIV-1 and subsequent biogenesis and release of EVs.

Analysis of EVs derived from HIV-1 infected cells has revealed the presence of various viral components, including the viral genome [23]. EVs derived from HIV-1 infected macrophages (Mɸs) and dendritic cells (DCs) can transfer infection to uninfected CD4+T-cells and induce robust viral replication [24, 25]. Moreover, recent studies have suggested EVs can migrate from the peripheral circulation to the CNS via crossing the BBB during inflammatory conditions and play a role in eliciting neuroinflammation [26–30]. Various potential mechanisms have been posited through which EVs can cross the BBB, including adsorptive mediated or clathrin mediated transcytosis and paracellular diffusion by breakdown of the tight junctions [28]. Studies in experimental models have shown that HIV-1 viral proteins sensitize HBMECs by inducing enhanced release of EVs containing gap junction proteins, facilitating infiltration of monocytes across a HBMEC monolayer [29].

During HIV-1 infection, BST-2 (bone marrow stromal antigen 2)/tetherin is upregulated and localized to virus-containing compartments (VCC) and attaches virions at the VCC-membrane interface [31]. BST-2 is a restriction factor of enveloped viruses including HIV-1, which inhibits virus release by tethering viral particles to the surface of infected cells [32–34]. Moreover, BST-2 is interferon-inducible, constitutively expressed in several cell types as a type II integral membrane glycoprotein, present in plasma membrane and intracellular compartments such as trans-Golgi network (TGN) and early endosomes [34–36]. BST-2 contains a short N-terminal cytoplasmic domain linked to a transmembrane region and a large extracellular domain attached to the membrane through a C-terminal glycosyl-phosphatidylinositol (GPI) moiety [35, 36]. BST-2 facilitates HIV-1 sorting through the endosomal compartment for eventual degradation in the lysosome [37, 38]. BST-2 knockdown alters VCC trafficking and promotes HIV-1 release, mediating its cell-cell transmission [31]. However, HIV-1 counteracts the antiviral activity of BST-2 by downregulating its expression at the cell surface by inducing its proteasomal degradation [34]. Recently, BST-2 has been shown to regulate EV release by tethering EVs to the cell surface [39]. Knocking down BST-2 in HeLa cells significantly reduced cell-surface associated EVs and concurrently enhanced their release [39].

Here, we investigated the effects of cocaine on the biogenesis and release of EVs in HIV-1 infected immune cells. Our results demonstrate that cocaine treated HIV-1 infected immune cells exhibit increased release of EVs; further, we found that these EVs mediated enhanced HIV-1 trans-infection and replication in T-cells. Analysis of the molecular mechanism of these phenomena revealed that cocaine downregulated the expression of BST-2 and altered intracellular trafficking of HIV-1 in DCs and macrophages, which resulted in enhanced incorporation of viral components into EVs. Further, we found that these EVs activated proinflammatory signals in HBMECs and altered permeability and integrity of its monolayer. Our results illuminate how EVs and cocaine can contribute to HIV-1 induced neuropathobiology and thereby enhance the prevalence of neurological disorders in cocaine using HIV-1 infected individuals.

## Results

### Cocaine enhances the release of EVs from HIV-1 infected immune cells and alters its molecular components

**Fig. 1:**
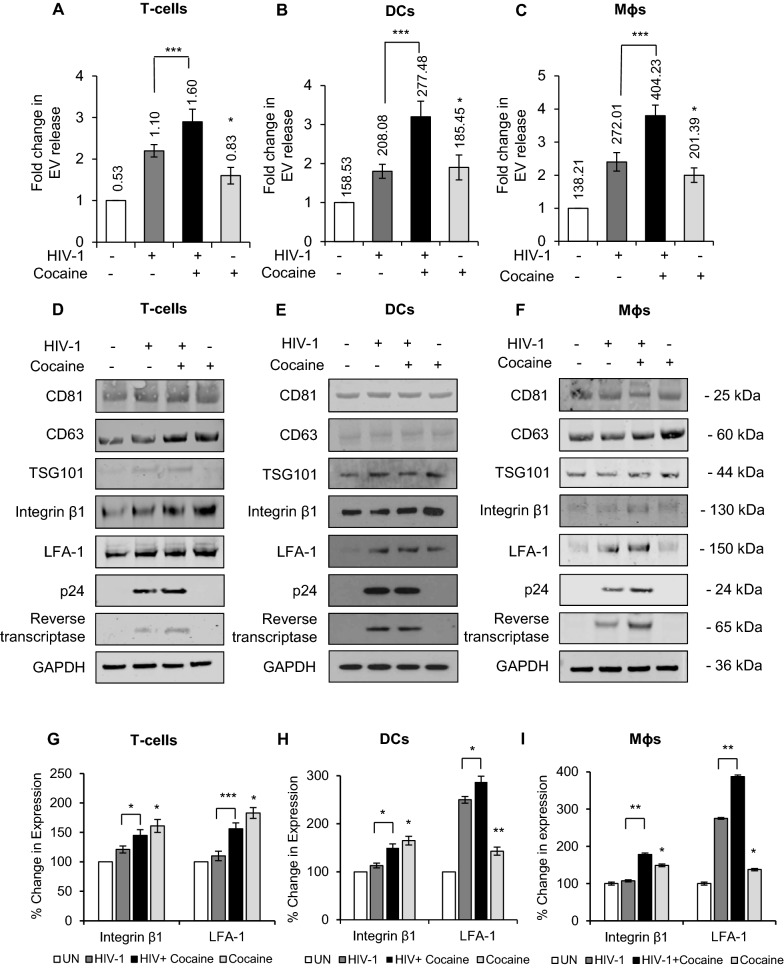
Cocaine alters the components and release of EVs in HIV-1 infected immune cells. **A**–**C** T-cells, DCs and macrophages were infected with 10 ng/ml HIV-1 and treated with or without 10 µM cocaine and EVs released were extracted by ultracentrifugation and quantified by NanoSight. Fold change was calculated with respect to the number of EVs released by uninfected, untreated cells. Data labels represent the absolute number of EVs released (× 10^9^ / ml). **D**–**F** EVs were extracted by ultracentrifugation, lysed and 15 µg of lysate protein were analyzed for the indicated molecular markers of EVs and HIV-1 proteins by Western blotting. GAPDH served as loading control. **G**–**I** Quantitative analysis of Integrin β1 and LFA-1 in D-F. The band intensity in each lane was determined by ImageJ software. The percent (%) change of each lane was determined by considering uninfected, untreated band as 100%. Data represent the mean ± SD of 3 independent experiments, and p-values were calculated relative to untreated controls (*p ≤ 0.05, **p ≤ 0.01, ***p < 0.001)

In order to investigate the effects of cocaine on the release of EVs from HIV-1 infected immune cells, we quantitated the EVs derived from untreated (control) (UN), HIV-1 infected (HIV-1), cocaine treated and HIV-infected (HIV-1+ Coc), cocaine alone (Coc) treated CD4+ T-cells (T-cells), monocyte derived dendritic cells (DCs), and monocyte derived macrophages (Mɸs). These immune cells were infected with 10 ng/ml HIV-1 after 2 hours of pretreatment with/without cocaine (10 µM); EVs were isolated by ultracentrifugation followed by immune-affinity (using anti-CD63 antibodies) purification, a published technique for EV isolation from virus infected cells [40]. Notably, EVs and HIV-1 share a common biogenesis machinery and other physicochemical properties, and thus, separation of both entities can be challenging. Hence, we identified our preparation as CD63+ extracellular vesicles (hereafter EVs). We observed significant increases in the number of EVs released by cocaine treated and HIV-1 infected (HIV-1+ Coc) DCs, Mɸs and T-cells (Fig. 1A–C) compared to untreated cells (UN). Further, we observed increased EV release in HIV-1+ Coc treated cells compared to HIV-1 infected cells. We then analyzed the molecular components of EVs by using Western blotting and observed the presence of EV markers in all samples (Fig. 1D–F). Upon quantitative analysis of western blots, we found significant upregulation of adhesion molecules Integrin β1 and LFA-1 (Integrin αL) (Fig. 1G–I).

We also analyzed these EVs for the presence of HIV-1 components including HIV-1 p24-Gag and Reverse Transcriptase by Western blotting. We observed the presence of these viral components in EVs derived from HIV-1 infected immune cells. Further analysis showed enhanced expression of HIV-1 p24-Gag and Reverse Transcriptase in EVs derived from HIV-1+ Coc Mɸs compared to HIV-1 Mɸs, however, this enhancement was not observed in HIV-1+ Coc T-cells and DCs (Fig. 1D–F).

### EVs derived from cocaine treated HIV-1 infected DCs and Macrophages show enhanced presence of viral genes and increased trans-infection of T-cells

**Fig. 2:**
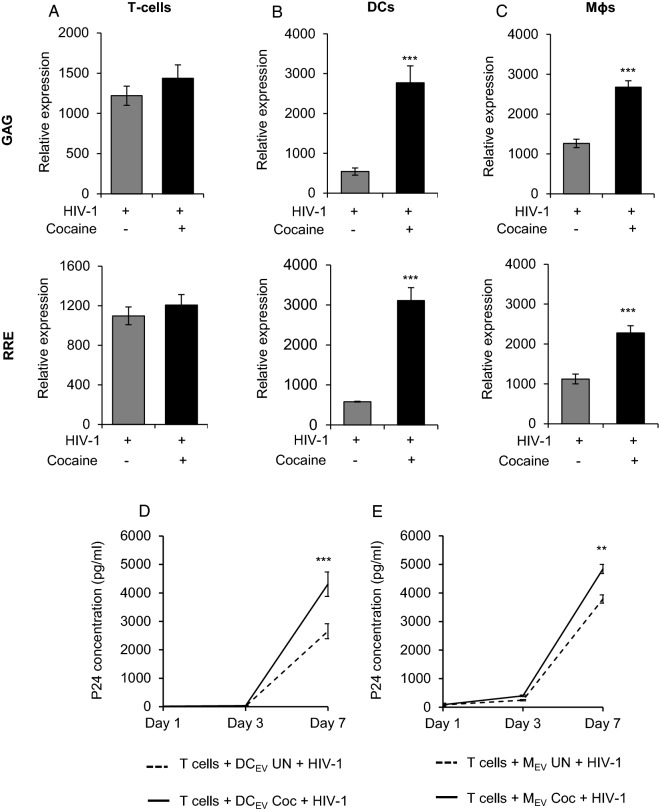
Cocaine enhances HIV-1 trans-infection mediated by EVs derived from DCs and macrophages. **A**–**C** Total RNA was isolated from EVs derived from HIV-1 infected, cocaine treated or untreated T-cells, DCs and macrophages. Expression of HIV-1 genes GAG (top panel) and RRE (lower panel) was analyzed by qRT–PCR. **D**–**E** EVs derived from HIV-1 infected, cocaine treated or untreated DCs (DC_EV_) and macrophages (M_EV_) were incubated with uninfected T-cells and the cell supernatant was analyzed for HIV-1 p24 titer on day 1, day 3 and day 7 after incubation by ELISA. Data represent the mean ± SD of 3 independent experiments, and p-values were calculated relative to untreated controls (*p ≤ 0.05, **p ≤ 0.01, ***p < 0.001)

Prior studies have shown that EVs derived from HIV-1 infected DCs and Mɸs carry viral molecules including viral genome and facilitate trans-infection [21, 25, 41, 42]. Consistent with these studies, our analysis of EVs derived from HIV-1 infected immune cells revealed the presence of viral genes (Fig. 2A–C). Intriguingly, when we analyzed the relative expression of HIV-1 genes GAG and RRE in these EVs, we found increased expression of both genes in EVs derived from HIV-1+ Coc DCs and Mɸs compared to those from HIV-1 DCs and Mɸs. However, we did not observe any significant changes in the expression of these viral genes in HIV-1+ Coc T-cells compared to HIV-1 T-cells (Fig. 2A–C). These results prompted us to investigate whether EVs derived from HIV-1 infected DCs and Mɸs can mediate trans-infection to T-cells. To address this question, fresh T-cells were incubated with EVs derived from HIV-1+ Coc or HIV-1 DCs and Mɸs and p24 titer was quantified in T-cells supernatants. Interestingly, while we observed viral replication in T-cells incubated with EVs derived from both HIV-1+ Coc and HIV-1 cells, the HIV-1 p24 titer was higher in supernatants of T-cells treated with EVs derived from HIV-1+ Coc DCs or Mɸs (Fig. 2D–E). These results indicated that cocaine treatment can enhance HIV-1 trans-infection to T-cells through EVs.

**Fig. 3:**
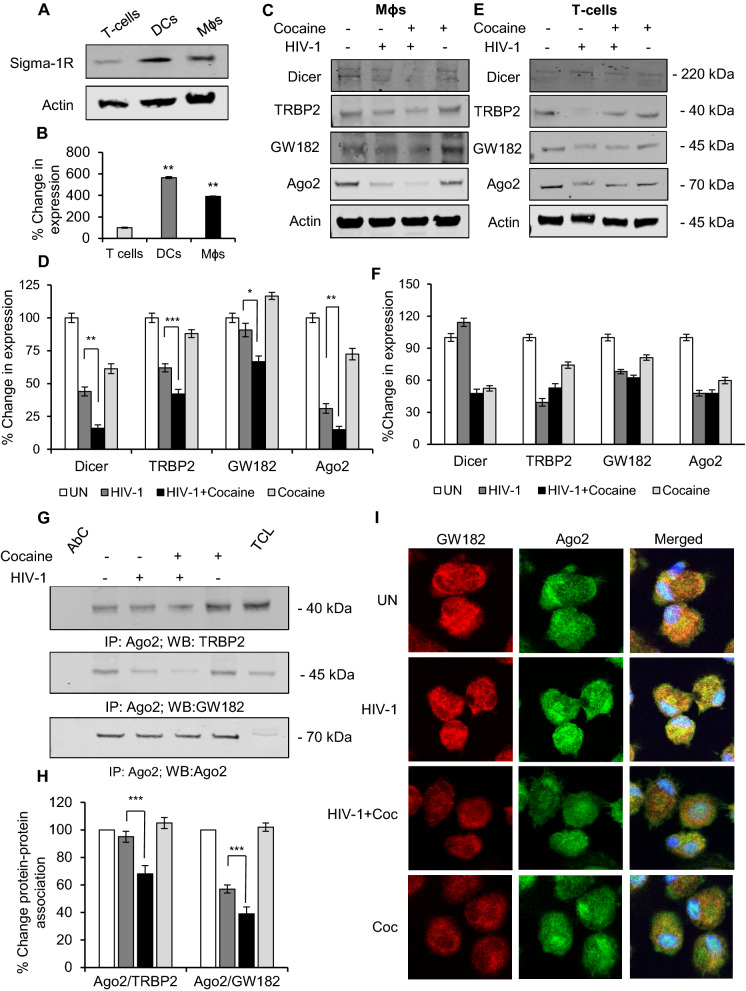
Cocaine treatment downregulates the RISC complex in macrophages. **A** Western blot analysis showing the expression of Sigma-1 receptor in untreated T-cells, DCs and macrophages. Actin served as loading control. **B** Quantitative analysis of the Western blots in (A). The band intensity in each lane was determined by ImageJ software. The percent (%) change of each lane was determined by considering untreated as 100%. **C** Western blot images showing expression of Dicer, TRBP2, GW182 and Ago2 in macrophages and **E** T-cells with or without HIV-1 infection and cocaine treatment. Actin served as loading control. **D**, **F** Quantitative analysis of the Western blots in (C, E) respectively. The band intensity in each lane was determined by ImageJ software. The percent (%) change of each lane was determined by considering untreated as 100%. **G** Ago2 immunoprecipitation and Western blot of TRBP2 and GW182 with Western blot of Ago2 as control. AbC—Antibody control; TCL—Total Cell lysate. **H** Quantitative analysis of the Western blots in (**G**). The band intensity in each lane was determined by ImageJ software. The percent (%) change of each lane was determined by considering untreated as 100%. **I** Representative confocal microscopic images showing interaction of Ago2 and GW182 in macrophages with or without HIV-1 infection and cocaine treatment after 6 days. Data represent the mean ± SD of 3 independent experiments, and p-values were calculated relative to untreated controls (*p ≤ 0.05, **p ≤ 0.01, ***p < 0.001)

Further characterizing the molecular mechanisms that may be involved in the differential effects of cocaine on T-cells, DCs and Mɸs, we initially examined the expression of the cocaine-associated receptor Sigma 1-receptor (Sig 1-R) in these cell types. Our data demonstrate that DCs and Mɸs express higher levels of Sig 1-R compared to T-cells (Fig. 3A, B), indicating that cocaine exerts a greater effect in Mɸs and DCs compared to T-cells likely due to receptor abundance.

Next, we investigated the effects of cocaine on RNA-Induced Silencing Complex (RISC) in these cell types by analyzing the expression of its components. RISC is known to be involved in gene silencing and mRNA degradation, and recently its role has also been implicated in intracellular trafficking of RNAs and its recruitment to EVs [43, 44]. The expression of components of RISC complex including Dicer, Argonaute-2 (Ago2), GW182 and TRBP2 were analyzed in UN, HIV-1, HIV-1+Coc and Coc treated Mɸs and T-cells by Western blotting. We observed significant downregulation of Dicer, Ago2, GW182 and TRBP2 in HIV-1 infected Mɸs and T-cells compared to uninfected cells (Fig. 3C–F). Further, when we analyzed the levels of expression among HIV-1+ Coc and HIV-1, we found significant decreases in the expression of these molecules in HIV-1+ Coc Mɸs compared to HIV-1 Mɸs. However, we did not observe this phenomenon in T-cells. Next, when we analyzed the interaction of Ago2 with GW182 or TRBP2 by immuno-precipitation, we found that interactions between Ago2 and GW182 or TRBP2 were reduced in HIV-1+Coc Mɸs compared to HIV-1 cells (Fig. 3G, H). Our confocal microscopic analysis also revealed decreased co-localization of GW182 and Ago2 in HIV-1+ Coc Mɸs compared to HIV-1 Mɸs (Fig. 3I). These findings suggest that downregulating RISC by cocaine might be a key mechanism underlying the enhanced recruitment of HIV-1 genes in EVs derived from Mɸs and DCs.

### Cocaine enhances release of EVs by downregulating BST-2 in HIV-1 infected cells

**Fig. 4:**
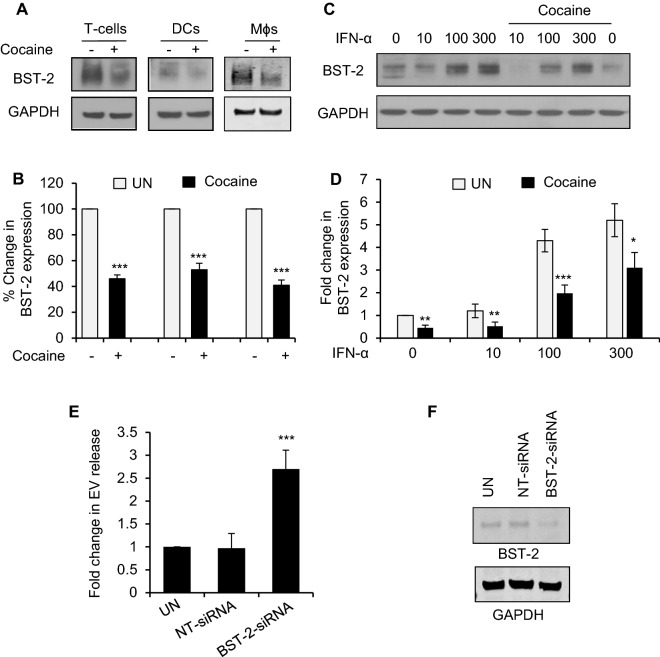
Cocaine enhances release of EVs by downregulating BST-2 in HIV-1 infected cells. **A** Western blot images showing BST-2 expression in T-cells, DCs and macrophages with or without cocaine treatment for 6 days. GAPDH was used as a loading control. **B** Quantitative analysis of the Western blots in (**A**). The band intensity in each lane was determined by ImageJ software. The percent (%) change of each lane was determined by considering untreated as 100%. **C** DCs were treated with increasing concentrations of IFN-α (0—300 IU/ml) and treated with or without 10 µM cocaine for 6 days were analyzed for BST-2 expression by Western Blotting. GAPDH was used as a loading control. **D** Quantitative analysis of the Western blots in (C). The band intensity in each lane was determined by ImageJ software. The fold change of each lane was determined by considering IFN-α untreated as reference. **E** U-937 cells were transduced with non-targeted siRNA or BST-2-siRNA and EV release was measured after 3 days by NanoSight. **F** Protein extracts from U-937 cells after transduction with NT-siRNA and BST-2-siRNA were analyzed for BST-2 expression by Western Blotting. Data represent the mean ± SD of 3 independent experiments, and p-values were calculated relative to untreated controls (*p ≤ 0.05, **p ≤ 0.01, ***p < 0.001)

In a previous study we observed that cocaine significantly downregulated BST-2, a Type II transmembrane protein known to offer intrinsic resistance against HIV-1 infection [21]. BST-2 or Tetherin cross-links viruses and EVs, tethering them to the plasma membrane [33, 39]. Hence, we analyzed the effect of cocaine on BST-2 expression in T-cells, DCs, and Mɸs. Cocaine downregulated the expression of BST-2 in all cell types (Fig. 4A, B). Since BST-2 is an interferon-inducible transmembrane protein, we tested whether cocaine inhibits enhanced expression of BST-2 by IFN-α in DCs. DCs were treated with increasing concentrations of IFN-α (0 - 300 IU/ml), with or without cocaine for 24 hours and analyzed for expression of BST-2. We found that cocaine significantly inhibited enhanced expression of BST-2 in DCs (Fig. 4C, D). To confirm whether the inhibition of BST-2 was responsible for enhanced release of EVs, we knocked down BST-2 using BST-2-siRNA in U937 cells and analyzed EV release. We observed a 2.7-fold increase in EV release when BST-2 was silenced in U937 cells compared to controls (Fig. 4E). We confirmed BST-2 silencing by Western blot analysis (Fig. 4F). These findings indicate that downregulation of BST-2 may be responsible for enhanced EV release in cocaine treated cells. Further, our electron microscopic analysis of BST-2 immuno-labelled HIV-1+ Coc DCs revealed enhanced release of EVs in these cells compared to HIV-1 infected or untreated cells. Electron microscopy images also showed decreased expression of BST-2 in the plasma membrane of HIV-1+ Coc cells (Additional file 1: Fig. S1B). Together, these findings support that cocaine can enhance the release of EVs from immune cells by downregulating BST-2.

### Cocaine alters BST-2 interaction with endosome biogenesis, ESCRT machinery, and intracellular trafficking

**Fig. 5:**
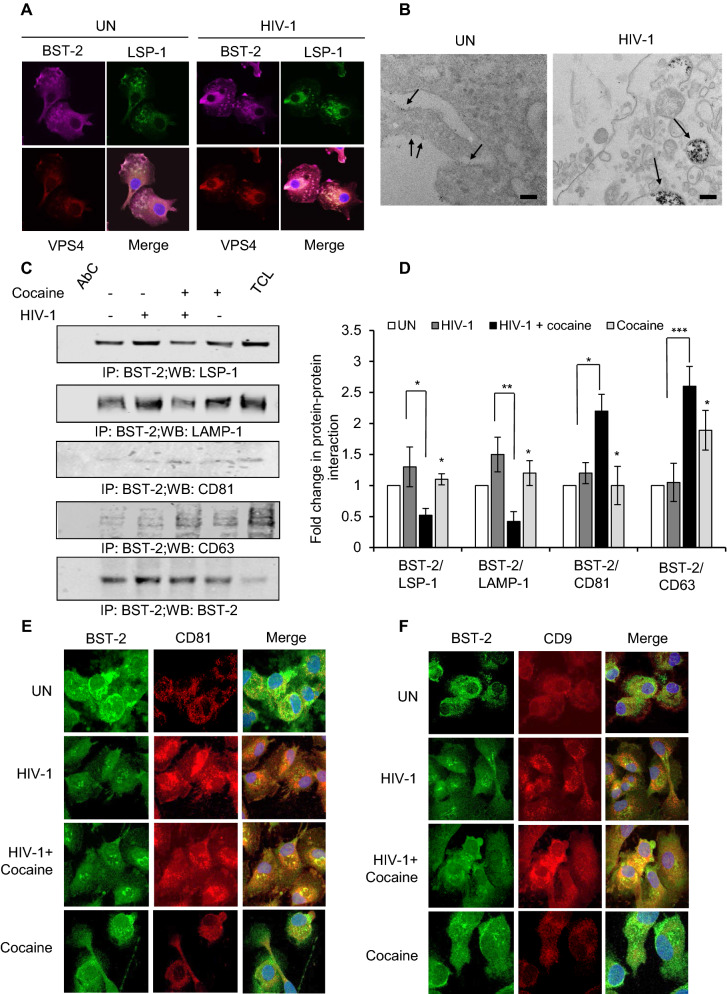
Cocaine treatment alters BST-2 interaction with intracellular trafficking, endosome biogenesis and ESCRT machinery. **A** Confocal microscopic image showing interaction of BST-2 with LSP-1 and VPS4 in macrophages with or without HIV-1 infection after 6 days. **B** Electron microscopic image showing immunolabelled BST-2 (indicated by black arrows) colocalization in macrophages with or without HIV-1 infection after 6 days. Scale bars = 200 nm. **C** BST-2 immunoprecipitation and Western blot of LSP-1, LAMP-1, CD81 and CD63 with Western blot of BST-2 as control. AbC—Antibody control; TCL—Total Cell lysate. **D** Quantitative analysis of Western blots showing fold change after HIV-1 infection and cocaine treatment in (**C**) with untreated and uninfected as control. The band intensity in each lane was determined by ImageJ software. **E** Confocal microscopic image showing interaction of BST-2 with CD81 and **F** CD9 in macrophages with or without HIV-1 infection and cocaine treatment after 6 days. Data represent the mean ± SD of 3 independent experiments, and p-values were calculated relative to untreated controls (*p ≤ 0.05, **p ≤ 0.01, ***p < 0.001)

BST-2, in addition to its functions at the cell membrane, is also known to anchor HIV-1 into the endosomal membrane and regulate intracellular trafficking of virus containing vesicles towards phagolysosomal complexes [45]. We thus investigated the interaction of BST-2 with components of intracellular trafficking and ESCRT machinery in HIV-1 infected cells. Confocal microscopic analysis revealed enhanced co-localization of BST-2 with Lymphocyte Specific Protein 1 (LSP-1) and Vacuolar protein sorting-associated protein 4 (VPS4), a member of the Endosomal Sorting Complexes Required for Transport (ESCRT) machinery, in HIV-1 DCs compared to uninfected cells (Fig. 5A). Next, EM analysis of BST-2 immunolabelled Mɸs revealed enhanced localization of BST-2 in endosomes of HIV-1 cells, indicating a role for BST-2 in intracellular trafficking of virus containing vesicles (Fig. 5B). Further, we studied the effects of cocaine on BST-2 mediated intracellular trafficking of virus containing vesicles in Mɸs. We analyzed the interaction of BST-2 with several intracellular trafficking molecules 24 hours post infection with or without cocaine. Immuno-precipitation experiments revealed decreased interaction of BST-2 with LSP-1 and with Lysosomal Associated Membrane Protein-1 (LAMP-1) in HIV-1+Coc cells compared to HIV-1 cells. We also observed enhanced co-localization of BST-2 with tetraspanins CD63 and CD81 in HIV-1+Coc cells compared to HIV-1 cells (Fig 5C, D). We confirmed these results by confocal microscopic analysis, which revealed enhanced co-localization of BST-2 with CD81 and CD9 in HIV-1+Coc Mɸs compared to HIV-1 cells (Fig. 5E, F). Overall, these results indicate that cocaine modulated BST-2 expression which altered the intracellular trafficking of intracellular vesicles and further led to the release of viral contents via EVs.

### EVs derived from cocaine treated HIV-1 infected Macrophages induced release of proinflammatory cytokines in Human Brain Microvascular Endothelial Cells (HBMECs) and altered endothelial barrier integrity and permeability

**Fig. 6:**
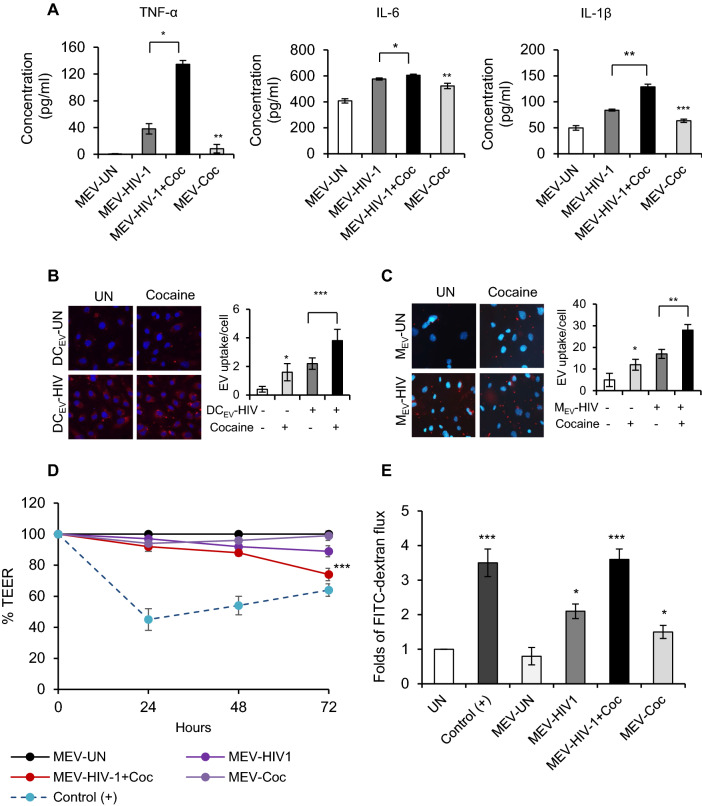
EVs derived from cocaine treated HIV-1 infected Macrophages induce inflammation and altered endothelial barrier integrity. **A** Quantitative analysis of TNF**-**α, IL-6 and IL-1β levels by ELISA in supernatants of Human Brain Microvascular Endothelial Cells (HBMECs) treated with EVs derived from cocaine treated or untreated, HIV-1 infected or uninfected macrophages. **B** Fluorescence microscopic image showing adhesion of PKH26-labelled cocaine treated or untreated, HIV-1 infected or uninfected DC derived and **C** macrophage derived EVs on HBMECs. Respective bar diagrams indicate number of EVs adhered to the HBMEC monolayer. **D** TEER was measured at indicated time intervals until 72 h after treatment with macrophage derived EVs by using Electric Cell-Substrate Impedance Sensing (ECIS) Ztheta 96 well array station. The impedance measurements were plotted as a percentage, with the reading of untreated as reference. **E** Fluorescence intensity of FITC-Dextran beads permeabilized through a HBMEC monolayer untreated or treated with 10 µM cocaine or treated with EVs derived from macrophages treated with or without cocaine, infected with or without HIV-1. VEGF was used as positive control. Data represent the mean ± SD of 3 independent experiments, and p-values were calculated relative to untreated controls (*p ≤ 0.05, **p ≤ 0.01, ***p < 0.001)

EVs are known to cross the BBB and induce neuroinflammation during inflammatory conditions and with HIV-1 infection [26–30]. To investigate the role of EVs derived from HIV-1+Coc immune cells in the elicitation of neuroinflammation, we analyzed the release of proinflammatory cytokines in Human Brain Microvascular Endothelial Cells (HBMECs) incubated with the EVs derived from UN, HIV-1, HIV-1+Coc and Coc Mɸs. Quantification of the cytokines TNF-α, IL-1β and IL-6 in the cell supernatants revealed significant increases in these cytokine levels after treatment with EVs derived from HIV-1 Mɸs compared to treatment with EVs derived from uninfected cells. In addition, cytokine levels were higher in cells treated with EVs derived from HIV-1+ Coc Mɸs compared to those treated with EVs from HIV-1 cells. EVs derived from cocaine treated macrophages (Coc Mɸs) also induced release of moderate levels of IL-1β and IL-6 in HBMECs (Fig 6A). Further, an EV uptake assay revealed EVs derived from HIV-1+ Coc Mɸs and DCs showed increased internalization into HBMECs compared to those from HIV-1 cells (Fig. 6B, C). We then analyzed effects of these EVs on HBMEC monolayer integrity and permeability. We found that EVs derived from HIV-1+ Coc Mɸs decreased Trans-Endothelial Electrical Resistance (TEER) and enhanced permeability of a HBMEC monolayer compared to a monolayer incubated with EVs derived from HIV-1 Mɸs (Fig. 6D, E). These findings demonstrate that EVs derived from HIV-1+ Coc infected Mɸs can both trigger release of proinflammatory cytokines in HBMECs and alter its monolayer integrity and permeability.

## Discussion

Extracellular vesicles (EVs) play a central role in intercellular communication and can contribute to various physiological and pathological processes [46]. Recent studies have implicated their participation in the pathogenesis of neuroinflammation and HIV-associated neurocognitive disorders (HAND) [26–30]. While, it has been suggested that EVs can migrate from the peripheral circulation to the CNS via crossing the BBB during inflammatory conditions [26–30], the role of EVs released from HIV-1 infected immune cells on elicitation of neuroinflammation has not been fully explored. Here, we sought to characterize the EVs derived from HIV-1 infected immune cells and study their effects on activation of proinflammatory molecules in HBMECs. Since recent studies have indicated a strong correlation between chronic cocaine abuse and HIV-1 associated neurological complications [3], we further addressed the effects of cocaine on biogenesis and release of EVs in HIV-1 infected immune cells and how the drug may augment neuroinflammation.

Our results show that cocaine treatment can significantly enhance the release of CD63+ EVs from both HIV-1 infected and uninfected immune cells. CD63 is a tetraspanin transmembrane molecule which also plays a role in the replication and production of HIV-1 [47]. Since CD63 is considered as one of the authentic and ubiquitously expressed markers for EVs, we identified this population as EVs.

Previous studies have shown that cocaine can enhance release of EVs from glioblastoma cells and human macrophages [22]. In analyzing the molecular composition of EVs derived from cocaine treated immune cells with or without HIV-1 infection, although we did not find significant differences in the expression patterns of major EV components, we defined enhanced expression of adhesion molecules such as integrin β1 and LFA-1 in EVs from cocaine treated cells. Cocaine treatment has been shown to enhance the expression of these adhesion molecules in various cell types, including immune cells. Intriguingly, we observed that cocaine treatment enhanced the recruitment of viral genes into EVs released from HIV-1 infected DCs and macrophages. However, we did not observe this phenomenon in CD4+ T-cells.

To address the molecular mechanisms mediating these effects of cocaine, we found that DCs and macrophages exhibited enhanced expression of the Sigma 1 receptor (Sig-1R) compared to CD4+ T-cells. Sig-1Rs are a unique class of non-G protein-coupled intracellular proteins known bind its ligands, including cocaine, and exert biological functions including regulation of transcription and function of various proteins [48]. The differential expression of Sig-1R in DCs and macrophages compared to T-cells may contribute to cocaine mediated effects in these cell types in terms of enhanced EV release and its altered cargo contents. Next, we analyzed the effects of cocaine on the RNA-induced silencing complex (RISC), multi-protein complex that contains dsRNA binding proteins, Argonaute (Ago) family of proteins, transactivation response RNA binding protein (TRBP), and Dicer, which processes pre-microRNAs into mature microRNAs (miRNAs) that target specific mRNA species [43]. The main functions of the RISC complex are gene silencing and mRNA degradation. Recently, the RISC complex has also been implicated in intracellular trafficking of RNAs and recruitment to EVs [44]. We found a significant decrease in expression these proteins in cocaine treated and HIV-1 infected DCs and macrophages. Notably, HIV-1 infection itself altered the integrity of the RISC complex, and cocaine further enhanced this effect. We did not observe significant changes in the RISC complex in CD4+ T-cells, even when infected with HIV-1. Since HIV-1 RNAs are not part of the Argonaute 2 associated RNA interference pathway in macrophages [49], we can hypothesize from our results that cocaine mediated disruption of the RISC complex may indirectly enhance cellular viral RNA and further its trafficking to EVs via suppressing the synthesis of HIV-1 RNA specific miRNAs.

BST-2, an interferon inducible intrinsic resistance factor of HIV-1, inhibits release of the virus by directly tethering virions to cells [31–35]. It also can regulate the release of EVs by anchoring them to the cell membrane [31–35]. Knocking down BST-2 in HeLa cells significantly reduced cell-surface associated EVs and concurrently enhanced their release [39]. Our studies revealed that cocaine mediated dramatic decreases in BST-2 which could contribute to the enhanced release of EVs from HIV-1 infected immune cells. Furthermore, we and others have shown that BST-2 plays a key role in the internalization of surface adhered viral particles and intracellular trafficking of virus containing vesicles [37, 38]. BST-2 physically anchors virions to intracellular vesicles and activates the ESCRT complex, which further facilitates their fusion with the phagolysosomal complex [37, 38]. We found that cocaine induced the downregulation of BST-2 and thus subverts intracellular transport of virion-containing vesicles by trafficking them towards an exosomal pathway rather than towards the phagolysosomal complex. Our study also revealed that cocaine induced downregulation of BST-2 can alter biogenesis, cargo recruitment and release of EVs in immune cells during HIV-1 infection.

Prior studies on EVs have highlighted another possible mechanism of retroviral spread and deregulation of the immune system via ‘Trojan exosomes’ [23, 25, 50–53]. Trojan EVs have been shown to possess a unique composition of both retroviral and host molecules and can transfer their contents to target cells independently of envelope protein-receptor interactions [50]. Intriguingly, such EVs also appear to play a role in the transmission of viral infection and elicitation of neuroinflammation by impairing the integrity of the BBB [25–30, 41]. Our study further revealed that incubation of EVs derived from cocaine treated HIV-1 infected macrophages induced release of proinflammatory cytokines in HBMECs and further altered integrity and permeability of its monolayer, indicating that such EVs may similarly alter the BBB and elicit neuroinflammation.

## Conclusions

**Fig. 7:**
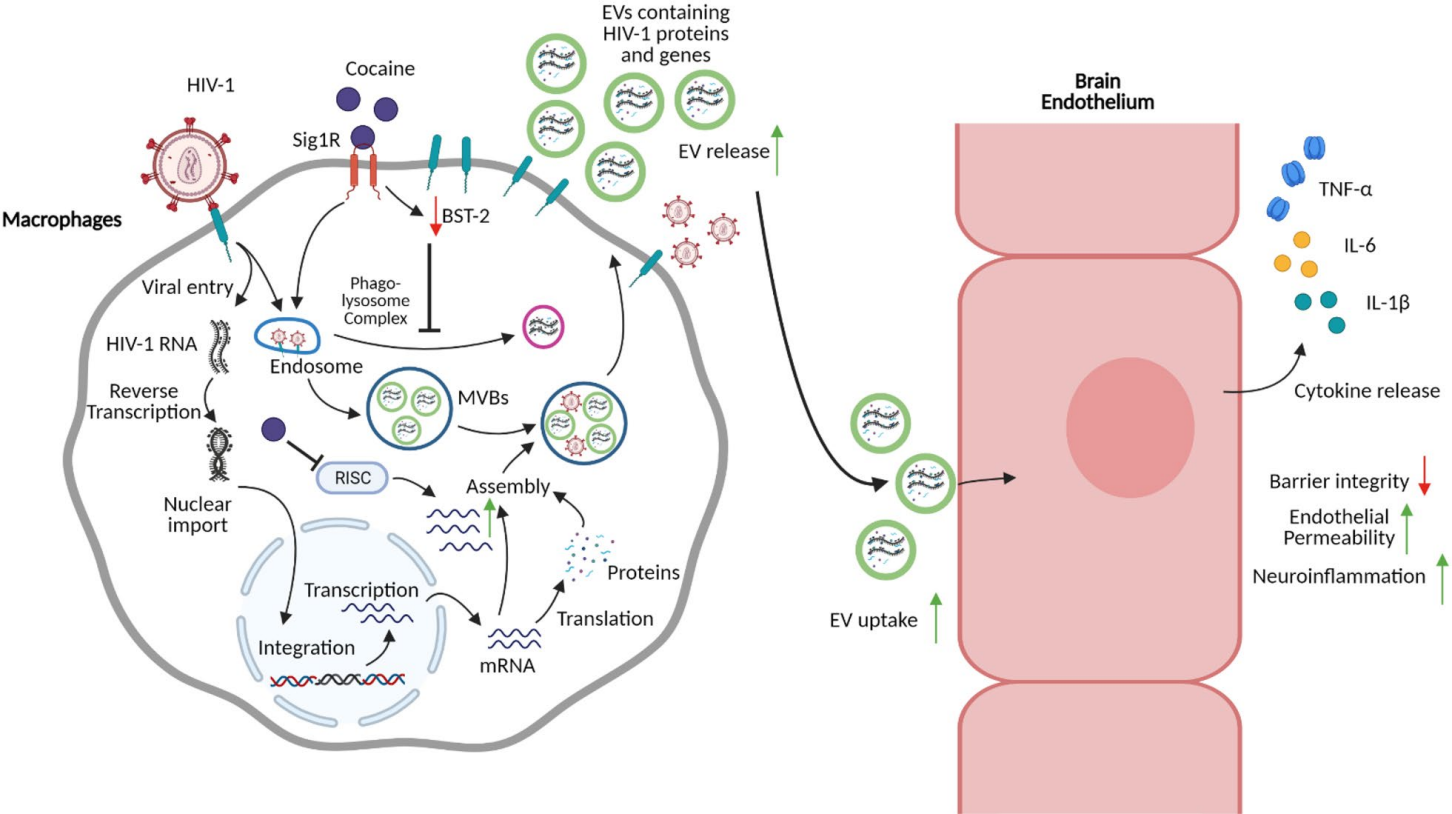
Schematic diagram showing cocaine enhancing neuroinflammation by increasing EV release in HIV-1 infected macrophages. Illustration created by Biorender.com

In sum, cocaine enhanced the release of EVs from HIV-1 infected DCs and macrophages. Characterization of these EVs revealed enhanced expression of adhesion molecules and significantly higher levels of viral genes. Further, we found that these EVs augmented viral trans-infection to CD4+ T-cells, triggered release of proinflammatory cytokines in HBMECs and altered monolayer integrity. Analyzing the molecular mechanism underlying these effects revealed that cocaine decreased the expression of BST-2, which resulted in the altered biogenesis, cargo recruitment and release of EVs. Our study thus elucidates how cocaine can be an important modulator of EVs from HIV-1 infected immune cells and contribute to their role in neuropathobiology. These results shed light on a novel mechanism which may enhance the prevalence of neurological disorders in cocaine using HIV-1 infected individuals and can provide insight into developing novel therapeutic strategies against HAND in these hosts. Figure 7 represents a schematic illustration of our study.

## Methods

### Cells, HIV-1 and constructs

Buffy coats were obtained from the Blood Transfusion Service, Massachusetts General Hospital, Boston, MA, in compliance with the Beth Israel Deaconess Medical Center Committee on Clinical Investigations (CCI) protocol #2008-P-000418/5. Buffy coats were provided at this institution for research purposes; therefore, no informed consent was further needed. In addition, buffy coats were provided without identifiers. This study was approved by Beth Israel Deaconess Medical Center’s CCI, Institutional Review Board, and Privacy Board appointed to review research involving human subjects. The experimental procedures were carried out in strict accordance with approved guidelines.

Human peripheral blood mononuclear cells (PBMC) were isolated from buffy coat by centrifugation, using a Ficoll-Paque density gradient (GE Healthcare Biosciences, Piscataway, NJ) and CD14+ monocytes were isolated using a positive selection kit per manufacturer’s protocol (STEMCELL Technologies, Inc., Vancouver, BC). Monocyte derived dendritic cells (hereafter referred as DCs) were prepared and cultured as previously described [21]. Monocyte-derived macrophages (hereafter referred as macrophages) were generated by culturing monocytes in RPMI supplemented with 10% FCS, 2 mM L-glutamine, 100 IU/ml penicillin, 100 μg/ml streptomycin, 1% nonessential amino acids, 1 mM sodium pyruvate, 500 IU/ml M-CSF (PeproTech Inc., Rocky Hill, NJ). Autologous T-cells from human peripheral blood mononuclear cells, were prepared and cultured as previously described [41]. Human Brain Microvascular Endothelial Cells (HBMEC) were kindly donated by Dr. Marsha Moses, Harvard Medical School, Boston, and cultured in Endothelial Basal Medium with growth supplements (Lonza, Alpharetta, GA) as per manufacturer’s instructions. HIV-1 infected U937 cells (U1) cells were obtained from NIH (Germantown, MD) and cultured in RPMI supplemented with 2 mM L-glutamine and 10% heat-inactivated FBS, as per instructions. HIV-1 replication was induced in U1 cells by treatment with 10 nM of Phorbol 12-myristate 13-acetate (PMA) (MilliporeSigma, Burlington, MA)

HIV-1 BaL was obtained from the NIH AIDS Research and Reference Reagent Program, National Institute of Allergy and Infectious Diseases, NIH. HIV-1 stocks were prepared as previously described [41].

### Antibodies and reagents

BST-2, CD63, CD9, CD81, TSG101, LSP-1, LAMP-1, GW182, HIV-1 Reverse Transcriptase, HIV-1 p24, Actin and GAPDH antibodies were obtained from Santa Cruz Biotechnology, Inc. (Santa Cruz, CA). Integrin β1, Ago2, TRBP-2 and Dicer antibodies were obtained from Cell Signaling Technology (Danvers, MA). MHC II antibody were obtained from Abcam (Cambridge, MA). LFA-1 antibody was obtained from R&D systems (Minneapolis, MN). Exosome-depleted FBS was obtained from System Biosciences (Palo Alto, CA).

### EV isolation

For exosome preparations, T-cells, DCs and macrophages (2 × 10^6^ cells/ml) were infected with HIV-1 BaL (10 ng/ml of HIV-1 p24) after 2 hours of pretreatment with/without cocaine (10 µM) in an exosome depleted medium. After 3 days, we quantitated the p24 titer in cell supernatants of all cells. EVs were isolated from cell supernatants by a combination of centrifugation and filtration: 700×*g* to remove cells and debris, filtering the supernatants on 0.45 µm pore filters, followed by ultracentrifugation at 100,000×*g* (Beckman L8-70M, Type 90 Ti Fixed-angle Titanium rotor and washing with 0.2 µm filtered 1X PBS by ultracentrifugation at 100,000×*g*. Next, CD63+ EVs were purified using Exo-Flow^™^ Exosome Purification kits (System biosciences, Mountain View, CA) or Exosome-Human CD63 Isolation/Detection Reagent from Invitrogen (Carlsbad, CA) as per manufacturer’s protocol. In each EV preparation, the concentration of total proteins was quantified by Bradford assay (Bio-Rad Laboratories, Hercules, CA). EVs were quantified by using Nano-flow cytometry and NanoSight. For experiments with RNA, pellets were lysed with RNA lysis buffer to isolate RNA. For experiments with EV lysates, pellets were lysed with 1x cell lysis buffer.

### Western blotting and immunoprecipitation

Western blotting was performed as previously described [21]. Briefly, EV pellets or whole cell pellets of uninfected and HIV-1 infected T-cells, DCs or macrophages were collected in cell lysis buffer, protein lysates were separated on NuPAGE precast gels (Life Technologies Corp.), transferred to 0.45 μm nitrocellulose membranes (Bio-Rad Laboratories, Hercules, CA), and probed with appropriate primary antibodies followed by incubation with their respective secondary antibodies (LI-COR, Lincoln, NE) and imaged using LI-COR Odyssey CLX (LI-COR). Membranes were stripped by using Re-Blot Plus (MilliporeSigma, Burlington, MA), and re-probed by using glyceraldehyde-3-phosphate dehydrogenase (GAPDH) or Actin as a loading control. Analysis and relative quantification of gel bands was carried out using ImageJ software (NIH, Bethesda, MD).

Immunoprecipitation assay was performed as previously described [21]. Equivalent amounts of protein extract were run on a 4% to 12% gradient acrylamide gel (NuPAGE Bis-Tris gel; Invitrogen, Carlsbad, CA) and transferred onto nitrocellulose membranes. Immunodetection involved specific primary antibodies, appropriate secondary antibodies conjugated to horseradish peroxidase, and a chemiluminescent Western blotting detection system (LI-COR).

### EV trans-infection in T-cells

T-cells (1 × 10^6^/ml) were infected either with 1 µg of total protein of CD63+ EVs derived from untreated or cocaine treated and HIV-1 infected macrophages and DCs for 7 days at 37°C. Supernatants were harvested on days 1, 3 and 7, and p24 concentrations were quantified by ELISA.

### Quantitative RT-PCR

RNA was isolated from EVs derived from cocaine treated or untreated HIV-1 infected T-cells, DCs and macrophages by Quick-RNA MiniPrep isolation kit according to the manufacturer’s instructions (Zymo Research, Irvine, CA). DNase treatment was performed using TURBO DNA-free kit (Ambion RNA, Carlsbad, CA). EV RNA (0.4 µg) was used to prepare cDNA using iSCRIPT cDNA synthesis kit (Bio-Rad, Hercules, CA). qRT-PCR was done in triplicates for each sample with SYBR green based SsoFast EvaGreen Supermix (Bio-Rad Laboratories, Hercules, CA) using 50 ng cDNA. Gene expression was normalized to TATA-box binding protein (TBP) and relative expression was calculated using 2−ΔCt method. Specificity of the primer sets was confirmed by melting curve analysis.

GAG_F: TTGGTCCAAAATGCGAACCC, GAG_R: ACTTGGCTCATTGCTTCAGC

RRE_F: TGGGCAAGTTTGTGGAATTGG, RRE_R: ACCTACCAAGCCTCCTACTATC

### Electron microscopy

DCs (1 × 10^6^) were cultured on Aclar (resin) coverslips. Cells were untreated or treated with cocaine (10 μM), infected or uninfected with HIV-1 (10 ng/ml) for 72 h. Cells fixed with 8% PFA 1:1 for 2 minutes followed by fixing in 4% PFA for 1 hour at 37 °C and replacing PFA with 1X PBS prior to immune-labelling. Subsequent processing and immunogold staining with anti-human BST-2 antibodies was done by Harvard Medical School Electron Microscopy Core.

### Confocal microscopy

Macrophages were cultured on chamber slides. They were untreated or treated with cocaine (10 μM), infected or uninfected with HIV-1 (10 ng/ml) for 72 h. They were fixed in 4% paraformaldehyde and blocked with 5% normal goat serum in PBS/Triton X100 (1 h). Cells were then incubated with primary antibodies overnight at 4 °C, washed thrice with PBS, and stained with AlexaFluor 546–labeled anti–mouse IgG antibody or AlexaFluor 488–labeled anti–rabbit IgG antibody (Molecular Probes®; Invitrogen) for 2 hours. Subsequently, cells were washed thrice with PBS, and slides were mounted using Prolong Gold antifade with DAPI (4′,6-diamidino-2-phenylindole; Invitrogen). Slides were examined under a Zeiss 880 Meta confocal microscope (Carl Zeiss Microimaging, LLC, Thornwood, NY), and images were acquired using ZEN2 software (Carl Zeiss).

### EV uptake assay

HBMECs were seeded into 8-well chamber slides at a density of 10^4^ cells per well and incubated in 5% CO_2_ incubator at 37°C for 24 hours to form a uniform monolayer of cells. The cells were treated with or without cocaine. After 30 minutes, the cells were treated with equal number of PKH26 labelled EVs derived from HIV-1 infected macrophages and DCs and incubated in 5% CO_2_ incubator at 37°C for 2 hours. Cells were washed for removal of unbound EVs and fixed with Formalin. The chamber slides were imaged using a wide-field fluorescence microscope (Carl Zeiss Microscopy LLC).

### Analysis of permeability of FITC-Dextran beads into the HBMECs monolayer

HBMECs were seeded into 24-well Transculture inserts with pore sizes of 0.4 µm (Corning Inc., Corning, NY) at a density of 10^5^ cells per well and incubated in 5% CO_2_ incubator at 37°C for 48 hours to form a tight monolayer of cells. The cell culture medium was replaced every 24 hours with fresh medium. The cells were treated with EVs derived from HIV-1 infected or uninfected cocaine treated or untreated macrophages and incubated for 72 hours. FITC-Dextran having a molecular weight of 70 kDa was then added to the apical chamber of each Transculture well and incubated for 30 minutes in 5% CO_2_ incubator at 37 °C. Permeability was analyzed by the endothelial transcellular passage of FITC-Dextran using fluorescence place reader.

### ELISA

Cell culture supernatants from different treatment and infection conditions were analyzed for TNF-α, IL-1β and IL-6 levels by corresponding ELISA kits according to the manufacturer's protocol (Chondrex, Inc., Redmond WA). P24 ELISA was performed using Zeptometrix ELISA kit according to the manufacturer's protocol (Zeptometrix Corporation, Buffalo NY). Supernatants were stored at −80°C.

### Trans-Endothelial Electric Resistance (TEER) Assay

Human Brain Endothelial Cells (HBMEC) were seeded into 96 wells (96W10idf PET, Applied Biophysics, Inc. Troy, NY) standard plate configuration containing two circular 350 μm diameter active electrodes on a transparent PET substrate (measuring from 100 to 200 cells) at a density of 5×10^4^ cells per well and incubated in 5% CO_2_ incubator at 37°C for 24 hours to form a uniform monolayer of cells. Cells were treated with or without 10 µM cocaine, infected with or without 10 ng/ml HIV-1. TEER was measured at various time intervals using Electric Cell-Substrate Impedance Sensing (ECIS) Ztheta 96 well array station (Applied BioPhysics, Inc.). Prior to the treatment the basal TEER was measured and confirmed the uniform resistance of monolayer in all the wells by measuring the TEER at 4000 Hz. TEER changes were calculated by taking TEER untreated monolayer as 100% at specific time point and calculated the changes in TEER of treated monolayer accordingly.

### Statistical analysis

All experiments were performed in triplicates. Differences between untreated and HIV-1 and/or Cocaine treated samples were calculated using a standard 2-tailed Student’s t-test. P-values ≤ 0.05 were considered statistically significant.

## Supplementary Information

**Additional file 1: Figure S1.** Cocaine enhances the release of EVs in HIV-1 infected macrophages and DCs. (**A**) Electron microscopy images of DCs infected with HIV-1 and treated with or without cocaine for 6 days and immune-labelled BST-2 (indicated by black arrows), scale bars = 200 nm. (**B**) Quantitative analysis of p24 and Reverse Transcriptase in Fig. 1F. The band intensity in each lane was determined by ImageJ software. The percent (%) change of each lane was determined by considering HIV-1 band as 100%. Data represent the mean ± SD of 3 independent experiments, and p-values were calculated relative to untreated controls (*p ≤ 0.05, **p ≤ 0.01, ***p < 0.001). **Figure S2.** Cocaine treatment alters the expression of components of RISC complex. Quantitative analysis of the colocalization of Ago2 and GW182 in macrophages, under conditions identical to Fig. 3I, using ImageJ2 software. Data represents mean of Pearson’s correlation coefficient indices of 10 randomly chosen images per condition (*p ≤ 0.05, **p ≤ 0.01, ***p ≤ 0.001, 2-tailed t-test). **Figure S3.** Cocaine modulates BST-2 expression by enhancing interaction with intracellular trafficking, endosome biogenesis and ESCRT machinery. (**A**) Quantitative analysis of the colocalization of BST-2 and LSP-1 in macrophages, under conditions identical to Fig. 5A, using ImageJ2 software. Data represents mean of Pearson’s correlation coefficient indices of 10 randomly chosen images per condition. (**B**) Quantitative analysis of the colocalization of BST-2 with CD81 and CD9 in macrophages, under conditions identical to Fig. 5E, F, using ImageJ2 software. Data represents mean of Pearson’s correlation coefficient indices of 10 randomly chosen images per condition (*p ≤ 0.05, **p ≤ 0.01, ***p ≤ 0.001, 2-tailed t-test).

## Data Availability

The data that support the findings of this study are available within the paper and its Additional file 1.

## Abbreviations

AIDS: Acquired immunodeficiency syndrome
BSA: Bovine serum albumin
BBB: Blood–Brain Barrier
BST-2: Bone marrow stromal antigen-2
CNS: Central Nervous System
DAPI: 4′, 6-Diamidino-2-phenylindole
DC: Dendritic Cell
ELISA: Enzyme linked immunosorbent assay
EM: Electron Microscopy
ESCRT: Endosomal sorting complexes required for transport
EV: Extracellular Vesicle
FBS: Fetal Bovine Serum
FCS: Fetal Calf Serum
GAPDH: Glyceraldehyde-3-phosphate dehydrogenase
GM-CSF: Granulocyte–macrophage colony-stimulating factor
GPI: Glycosyl-phosphatidylinositol
HAND: HIV-1 associated neurological disorder
HIV-1: Human Immunodeficiency Virus 1
IFN-α: Interferon-α
LFA-1: Lymphocyte function-associated antigen-1
MHC II: Major histocompatibility complex II
MVB: Multivesicular bodies
PBMC: Peripheral Blood Mononuclear Cells
PBS: Phosphate-buffered saline
PFA: Paraformaldehyde
PMA: Phorbol 12-myristate 13-acetate
RISC: RNA-induced silencing complex
Sig-1R: Sigma-1 Receptor
siRNA: Small interfering RNA
TEER: Trans-endothelial electrical resistance
TGN: Trans-Golgi network
TRBP2: Trans-activation responsive RNA binding protein 2
VCC: Virus-containing compartment
