# Supplementary figures and images for: Cocaine augments neuro-inflammation via modulating extracellular vesicle release in HIV-1 infected immune cells
Source: Retrovirology. 2021 Sep 16;18:26. doi: 10.1186/s12977-021-00570-4 (PMC8444590; doi:10.1186/s12977-021-00570-4)

Supplementary Figure 1

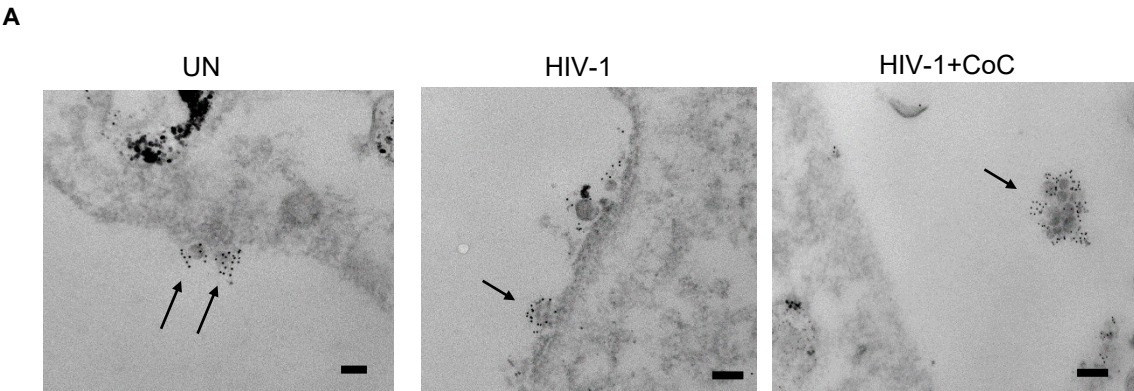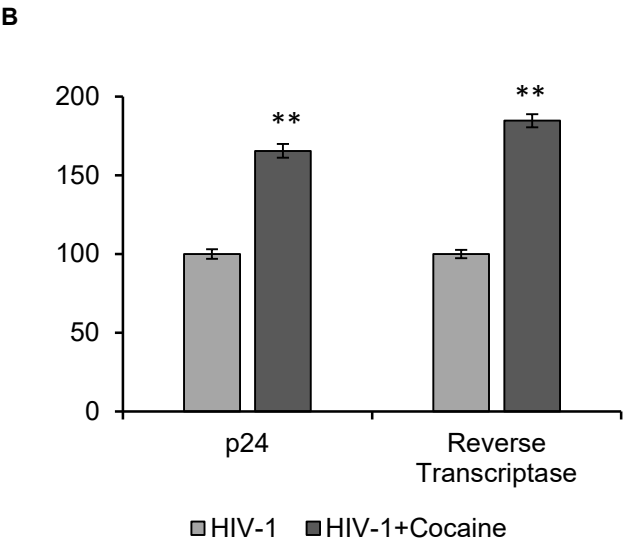

Supplementary Figure 2

A

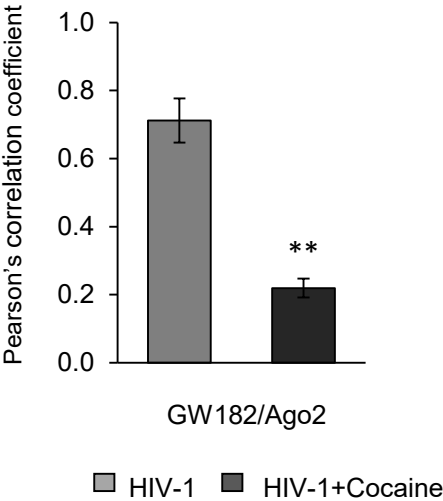

Supplementary Figure 3

A

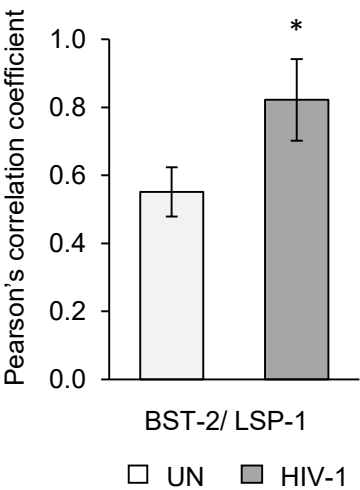

B

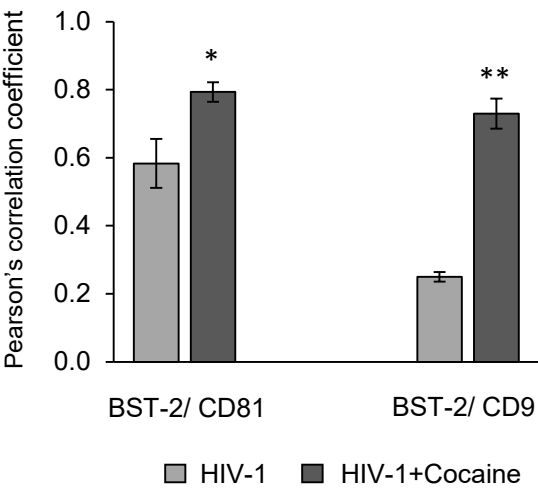

Supplement: Supplementary file 1 — Additional file 1: Figure S1. Cocaine enhances the release of EVs in HIV-1 infected macrophages and DCs. (A) Electron microscopy images of DCs infected with HIV-1 and treated with or without cocaine for 6 days and immune-labelled BST-2 (indicated by black arrows), scale bars = 200 nm. (B) Quantitative analysis of p24 and Reverse Transcriptase in Fig. 1F. The band intensity in each lane was determined by ImageJ software. The percent (%) change of each lane was determined by considering HIV-1 band as 100%. Data represent the mean ± SD of 3 independent experiments, and p-values were calculated relative to untreated controls (*p ≤ 0.05, **p ≤ 0.01, ***p < 0.001). Figure S2. Cocaine treatment alters the expression of components of RISC complex. Quantitative analysis of the colocalization of Ago2 and GW182 in macrophages, under conditions identical to Fig. 3I, using ImageJ2 software. Data represents mean of Pearson’s correlation coefficient indices of 10 randomly chosen images per condition (*p ≤ 0.05, **p ≤ 0.01, ***p ≤ 0.001, 2-tailed t-test). Figure S3. Cocaine modulates BST-2 expression by enhancing interaction with intracellular trafficking, endosome biogenesis and ESCRT machinery. (A) Quantitative analysis of the colocalization of BST-2 and LSP-1 in macrophages, under conditions identical to Fig. 5A, using ImageJ2 software. Data represents mean of Pearson’s correlation coefficient indices of 10 randomly chosen images per condition. (B) Quantitative analysis of the colocalization of BST-2 with CD81 and CD9 in macrophages, under conditions identical to Fig. 5E, F, using ImageJ2 software. Data represents mean of Pearson’s correlation coefficient indices of 10 randomly chosen images per condition (*p ≤ 0.05, **p ≤ 0.01, ***p ≤ 0.001, 2-tailed t-test). [file 12977_2021_570_MOESM1_ESM.pdf]
